# Pluripotent Stem Cells for Retinal Tissue Engineering: Current Status and Future Prospects

**DOI:** 10.1007/s12015-018-9802-4

**Published:** 2018-04-19

**Authors:** Ratnesh Singh, Oscar Cuzzani, François Binette, Hal Sternberg, Michael D. West, Igor O. Nasonkin

**Affiliations:** BioTime, Inc., 1010 Atlantic Avenue, Alameda, CA 94501 USA

## Abstract

The retina is a very fine and layered neural tissue, which vitally depends on the preservation of cells, structure, connectivity and vasculature to maintain vision. There is an urgent need to find technical and biological solutions to major challenges associated with functional replacement of retinal cells. The major unmet challenges include generating sufficient numbers of specific cell types, achieving functional integration of transplanted cells, especially photoreceptors, and surgical delivery of retinal cells or tissue without triggering immune responses, inflammation and/or remodeling. The advances of regenerative medicine enabled generation of three-dimensional tissues (organoids), partially recreating the anatomical structure, biological complexity and physiology of several tissues, which are important targets for stem cell replacement therapies. Derivation of retinal tissue in a dish creates new opportunities for cell replacement therapies of blindness and addresses the need to preserve retinal architecture to restore vision. Retinal cell therapies aimed at preserving and improving vision have achieved many improvements in the past ten years. Retinal organoid technologies provide a number of solutions to technical and biological challenges associated with functional replacement of retinal cells to achieve long-term vision restoration. Our review summarizes the progress in cell therapies of retina, with focus on human pluripotent stem cell-derived retinal tissue, and critically evaluates the potential of retinal organoid approaches to solve a major unmet clinical need—retinal repair and vision restoration in conditions caused by retinal degeneration and traumatic ocular injuries. We also analyze obstacles in commercialization of retinal organoid technology for clinical application.

## Background

Human pluripotent stem cells (hPSCs) possess two key intrinsic properties that distinguish them from all other cell types. First, they display the potential to differentiate into all somatic cell lineages and some extraembryonic tissues [1–4] and even self-organize into developing embryonic tissue *anlagen* (primordia) [5–8]. Second, they show replicative immortality while maintaining long telomeres [9, 10], making them a reliable and replenishable source of cells for differentiation and translational research. These properties open the door to a host of potential therapeutic strategies for many devastating diseases caused by genetic conditions, trauma or simply aging. In less than two decades, facile methods of reprogramming fully differentiated somatic cells back to a pluripotent state have become widely implemented [11, 12]. Leveraging the replicative immortality of hPSCs strategies have been developed for the targeting of the genome to engineer precise genetic modifications [13]. Lastly, a growing understanding of the gene regulatory networks and epigenetic basis of differentiation provide a new highly sophisticated picture of how a human cell acquires and maintains a specific cell fate. These and other recent advances enable the design of novel protocols for the engineering of cells of different lineages in a dish, using hPSCs or even terminally differentiated cells as a starting material. The three-dimensional tissues (organoids) grown in a dish are developmentally, anatomically and physiologically similar to tissues and organs developed in vivo [8]. Such ability has huge implications for translational medicine, since these cells have been implicated for use in cell replacement, disease modeling and drug screening.

Among the stem cell replacement therapies, retinal stem cell therapy stands out as a low hanging fruit, because it is one of the most urgent unmet needs, and technically the most feasible one. The eye is a small, encapsulated organ, with simple neuroanatomy and privileged immune status [14]. The ocular space is easily accessible for transplantation and retinal grafts can be easily visualized using noninvasive methods. Millions of people around the world suffer from retinal degenerative diseases such as Age-related macular degeneration (AMD), Retinitis pigmentosa (RP) and Stargardt’s disease (SD) that lead to permanent vision loss. Blindness is costly and is a major burden on our society [15–18]. At present, there is no satisfactory treatment available for these disorders; hence, it is essential to develop more effective treatments as well as preventive methods. The ability of hPSCs to form retina in a dish [19] is being explored to develop new vision restoration strategies, based on replacing hPSC-derived retinal tissue rather than individual types of retinal cells [20–22]. The knowledge of neuroanatomical structure and connectivity of human retinal tissue supports this approach, and preexisting accumulated technology of retinal replacement [23] may help to transform this leap forward in thinking into urgently needed therapy.

In this review, we discuss structure and function of retina, sources of stem cells for derivation of three dimensional (3D) retinal tissue, potential challenges in retinal transplantation, alternative methods of retinal tissue engineering and challenges in commercializing retinal organoid technology for clinical applications.

## Anatomy and Function of Retina

**Fig. 1:**
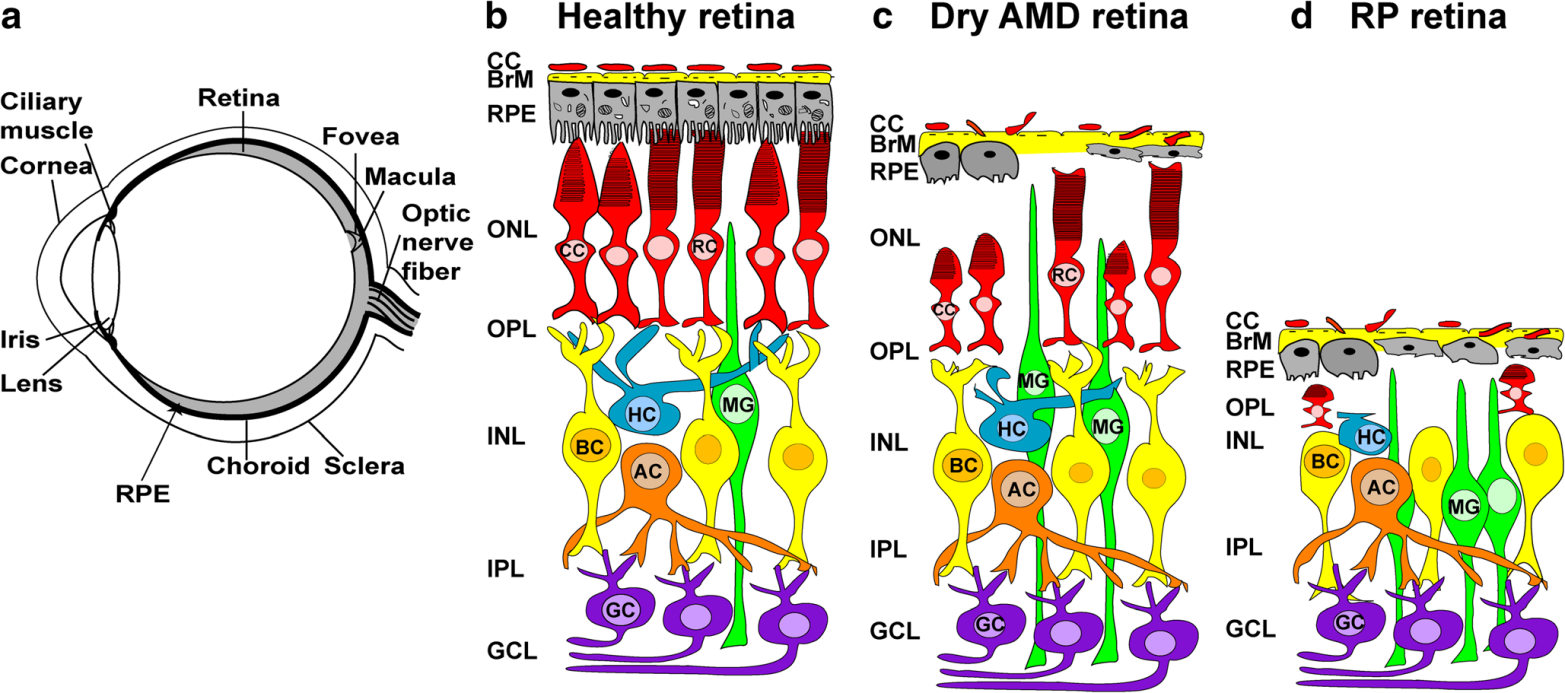
**(a**) Schematic drawing of a cross-section through a human eye. Light enters the eye through the cornea, passes through the pupil, lens and strikes the retina. Retina is the light-sensitive tissue lining the inner surface of the eye. Visual information from retina transmits to the brain through optic nerve fiber. In the middle of the retina there is a small depression called fovea, which is responsible for high-resolution vision. Region surrounding the fovea is called macula and is rich in cones. Retinal pigment epithelium (RPE) is a pigmented cell layer separating the choroidal blood supply from the photoreceptor layer. Choroid is a vascular layer of the eye. Sclera is a tough white sheath around the outside of the eye ball. **(b**) Schematic diagram of healthy retinal circuits. Mammalian retina consists of six major types of neuronal cells – rod cells (RC) and cone cells (CC), horizontal cells (HC), bipolar cells (BC), amacrine cells (AC) and retinal ganglion cells (RGC). The Müller cell (MC) are the glial cell that span across the retina and their somata. RPE provides metabolic and transport functions essential for homeostasis of the neural retina. Bruch’s membrane (BrM) is a highly specialized and multi-laminar structure separating RPE from the choroid and mediates exchange of oxygen and nutrients between vasculature of choroid and neural retina. RPE and the Bruch’s membrane form the outer blood–retinal barrier. Choroidal capillaries (CC) are the blood capillaries present in choroid that supply oxygen and nourishment to the outer layer of the retina. **(c**) Schematic diagram of dry age-related macular degeneration (AMD) retinal circuit. In Dry AMD, there is progressive atrophy of retinal pigment epithelium (RPE), Bruch’s membrane (BrM) and choriocapillaris (CC) in the macula. As a result, RPE cells stop providing support functions and the photoreceptors in the macula die, resulting in a loss of central vision. **(d**) Schematic diagram of retinitis pigmentosa (RP) retinal circuit: In RP, rod photoreceptors die, which trigger dramatic changes in the morphology of second order neurons (horizontal cells, bipolar cells and amacrine cells). As a result of the rapid rod degeneration, rod-driven bipolar and horizontal cell axon terminals retract their fine dendrites, and rod bipolar cell axon terminals assume immature synaptic structures. Defects extend to the cone circuit during the late phase of degeneration. In this case, both cones and cone horizontal cells sprout new neurites, whereas cone bipolar cells retract their dendrites

The retina is the photosensitive component of the central nervous system (CNS), lining the inner surface of the eye (Fig. 1a). It consists of five types of neuronal cells: photoreceptor cells (rods and cones), horizontal cells, bipolar cells, amacrine cells, ganglion cells and support cells (Müller glia cells) (Fig. 1b) [24–31]. Retinal neurons are organized into three distinct nuclear layers, which are separated by two synaptic layers [32–34]. The nuclei of the rod and cone photoreceptors form the outer nuclear layer (ONL), the nuclei of horizontal cell, bipolar cells and amacrine cells form the inner nuclear layer (INL), and the innermost nuclear layer contains ganglion cells and a few astrocytes (glial cells) and is called the ganglion cell layer (GCL). The photoreceptors establish synaptic contacts with horizontal cells and bipolar cells in the first synaptic layer, the outer plexiform layer (OPL). In the second synaptic layer, the inner plexiform layer (IPL), bipolar and amacrine cells make synaptic contacts with ganglion cells [35]. The Müller cells are glial cells that span across the retina and provide support structure stretching radially across the full thickness of the retina [36–40]. Adjacent to the photoreceptor layer is a layer of pigmented epithelial cells, called the retinal pigment epithelium (RPE) [41–46], which are essential for maintenance of rod and cone photoreceptor cells (Fig. 1b). The apical surface of the RPE has microvilli, which interact with the photoreceptor outer segments [47, 48]. The basolateral surface of RPE is attached to the underlying layer of Bruch’s membrane (BrM), which separates the RPE from the endothelium of the choriocapillaris (CC) [49].

During development six different neuronal cell types and a single glial cell type, develop in a sequential order from a pool of cycling multipotential retinal progenitors [27, 50]. Retinal ganglion cells develop first, closely followed by horizontal cells, cones and amacrine cells, while rod photoreceptors, bipolar and Müller glia cells are generated in the latter half of retinogenesis [50]. Photoreceptors convert light into an electrical signal that pass through the second and third order neuron to the brain via the optic nerve. These cells are critically important for vision, as the initial capture of visual information (photons) takes place only in photoreceptors. Photoreceptor cell death inevitably leads to blindness. Photoreceptors receive trophic and structural support from RPE cells [47, 48, 51–53]. In dry AMD, disruption of the RPE causes accumulation of drusen (waste products from retinal photoreceptor cells inside and below the RPE cells) [54, 55]. Accumulation of drusen contributes to addition of complement components and other acute phase proteins, leading to a low level pro-inflammatory macrophage response and eventual subretinal neovascularization. In the advanced stage of dry AMD, RPE loss causes thinning of the cone photoreceptors in the macula leading to atrophy, or cell death (Fig. 1c) [56–58]. Likewise, in RP rod photoreceptor cell death triggers secondary cone degeneration, even though the specific mutation affects only rods but not cones (Fig. 1d) [59–61]. Once the photoreceptors start to die, their synaptic partners (rod and cone bipolar neurons in the inner nuclear layer) initiate synaptic remodeling, where rod bipolar neurons synapse on cone photoreceptors in the absence of rods [62]. Eventually, when the majority of photoreceptors die, the loss of synaptic partners triggers bipolar cell death (“death by trophic deprivation”) [63], precipitating additional remodeling and eventual death of other types of neurons in the inner nuclear layer (anterograde trans-synaptic degeneration) [62]. However, RGCs remain seemingly unaffected and lack apoptotic signals even when the ONL and the INL degenerate as long as there is preservation of axonal connectivity with the neurons of the visual cortex [64, 65]. RGCs receive and require continuous trophic support from their synaptic partners in the brain; the optic nerve transection is a classical model of axotomy and neurodegeneration and leads to RGC cell death [66–70].

## Retina: Ideal Target Tissue for Stem Cell Based Therapy

The retina is a very fine and layered neural structure, which vitally depends on preservation of cells, neural anatomy and synaptic networks in order to maintain vision. Preserving or restoring the original neural architecture of retina and photoreceptor-second order retinal neuron connectivity is a major therapeutic goal to alleviate blindness in millions of people worldwide caused by RP and AMD [35, 71]. The eye is a small organ; and the number of stem cells required for a therapeutic procedure would be theoretically lower than with larger organs. The retinal environment can be easily accessed with small gauge vitrectomy needles compared to other internal organs, which greatly increases the potential of stem cell based therapy for treatment of retinal degenerative diseases. Stem cell-derived RPE cells or retinal progenitor cells were successfully transplanted into the subretinal space, which limits the systemic circulation of young cells and circumvents the immune rejection [72, 73]. The eye, in addition to the brain, is a relatively immune privileged organ [74–76], where the immune privileged environment is provided by immunosuppressive and anti-inflammatory factors present in the ocular tissue and fluids [77]. Examples of such factors include transforming growth factor β2 (TGF β2), Fas ligand, complement regulatory proteins (CRP), macrophage migration inhibitory factor (MIF), alpha-melanocyte stimulating hormone (α-MSH) and systemic form of tolerance to the foreign antigen called anterior chamber associated immune deviation (ACAID). In the eye, an immunosuppressive milieu is created by the blood-retinal barrier (BRB) consisting of RPE (outer BRB) and the endothelium of the inner retinal microvasculature (inner BRB, analogous to blood brain barrier). Streilein et al. further classified the levels of retinal immune privilege as “full immune privilege” (in the subretinal space, including RPE) and “partial immune privilege” (in the neural retinal tissue, which is vascularized) [74].

## Source of Cells for Retinal Transplantation

### Fetal Retinal Progenitor Cells and Tissue

Fetal derived retinal progenitor cells (RPCs) are multipotent cells that can give rise to all six neuronal types of retinal cells and Müller glia. Retinal progenitor cells display immature markers such as PAX6, VSX2, LHX2, SIX3, RAX indicative of retinal stem cell fate [78–84] and can be used for successful subretinal transplantation [78, 85, 86]. Several studies done in animal models demonstrated that subretinal transplantation of retinal progenitor cells derived from human or mouse fetal or neonatal retina can rescue photoreceptor function and leads to maturation of RPCs and expression of Rhodopsin marker [78, 85, 87–90]. However, major disadvantage of using fetal RPCs is generating sufficient number of donor cells for transplantation. A second disadvantage is limited functional (synaptic) integration of donor cells. Although some improvements in visual function were reported in animal models with retinal degenerative diseases [78, 85, 91, 92], this could be partially attributed to trophic factor (neuroprotective) support from grafted cells, rather than changes caused by specific cell replacement [93]. However, advanced testing in retinal degenerative (*rd*) mice with mouse photoreceptor progenitor grafts discovered formation of specific graft-host synaptic connectivity and activation of visual areas in the brain, specifically primary visual cortex (V1*)* [94]. This indicates a cell replacement-mediated recovery mechanism is involved in vision improvement rather (or, in addition to) neuroprotection. Currently, California - based regenerative medicine company jCyte. Inc, has completed phase1/2a clinical trial (#NCT02320812) (Table 1) to study the safety of hRPCs in 28 RP patients. Early results were promising and revealed no significant side effects, with good tolerance of injected cell. The limited therapeutic impact from these cells, injected into the vitreous, is clearly due to their neuroprotective function only. ReNeuron. Inc, is also conducting a clinical trial in advanced RP patients using hRPCs. The trial is designed to assess safety, tolerability and preliminary efficacy of the hRPCs in RP patients (NCT02464436) (Table 1). Based on positive results from their preclinical animal work [85], positive results in patients may also be expected, but the likely rescue mechanism is neuroprotective rather than cell replacement.

**Table 1:** Clinical trials using human fetal tissue and HESCs

Disease	Source	Phase	Clinical trial.gov identifier	Cell type transplanted	Center (PI)
Retinitis Pigmentosa	Human fetal tissue	I/II	NCT02464436	Retinal progenitor cell	ReNeuron, Guildford, UK
Retinitis Pigmentosa	Human fetal tissue	I/II	NCT02320812	Human retinal progenitor cell	jCyte, Inc, CA USA
Retinitis Pigmentosa	Human fetal tissue	Phase II	NCT03073733	Human retinal progenitor cell	jCyte, Inc, CA USA
AMD	Human fetal tissue	Phase II	NCT00346060	Human fetal retinal sheet	Kentucky, USA
Retinitis Pigmentosa	Human fetal tissue	Phase II	NCT00345917	Human fetal retinal sheet	Kentucky, USA
Stargardt’s Macular Dystrophy	HESC	Phase I/II	NCT01345006	RPE	Astellas, USA
Dry AMD	HESC	Phase I/II	NCT01344993	RPE	Astellas, USA
AMD	HESC	Phase I/II	NCT02286089	RPE	Cell Cure, Israel
Dry AMD	HESC	Phase I/II	NCT02590692	CPCB-RPE1	Regenerative Patch Technologies, LLC, CA, USA

An alternative approach used for vision treatment was transplanting fetal retinal sheet in the subretinal space. Studies in animal models demonstrated that transplantation of a fetal retinal sheet with or without RPE can repair damaged retina. These sheets of cells develop like a normal retina, in contrast to single cell suspensions, and release trophic support to the host retina [95, 96]. However, survival of fetal tissue depends on several conditions: fetal tissue should be immature, retinal surgery is done without damaging retinal vasculature, retinal sheet is placed parallel to the recipient RPE enabling the graft to interact with recipient retinal niche, neural retina and RPE are transplanted as a single sheet, and retinal ganglion cells of the recipient retina are preserved [23]. The importance of grafting young rather than adult retinal tissue was elaborated in work by Aramant and Seiler [97], who demonstrated that retinal donor age has a strong impact on the outcome of retinal transplantation. Fetal retinal tissue is typically undergoing lamination and retinal cell type fate commitment when harvested for grafting, yet is not mature enough to be compromised due to disruption of axons or neurons during subretinal transplantation surgery. This allows preservation of the primordial retinal structure in human embryonic retinal tissue, which is instrumental for priming further development of photoreceptors and second order neurons to mature retinal tissue in subretinal grafts [95]. Such structure is destroyed during dissociation of mammalian fetal retina to progenitors and cannot reassemble back into laminated retinal tissue [98]. The demonstration of the importance of full retinal structure preservation is based on a report by Ghosh et al., who attempted to transplant vibratome-sectioned rabbit fetal and adult retina by removing INL and RGCs, and reported that this approach failed while transplanting “full-thickness” embryonic retina was successful [99]. Accordingly, the overarching therapeutic idea is that embryonic/fetal retinal tissue has the potential to (i) complete differentiation in subretinal space, (ii) synapse on the recipient retinal neurons and (iii) establish connectivity with the visual cortex. In addition, adult human RPE cells were also used as a source of cells for retinal dystrophy therapies [100–102], where the patient’s own RPE cells degenerate (e.g., AMD). Similar to human fetal RPCs, the limited number of cells may be a problem for cell therapy applications using human primary RPE [102]. However, innovative approaches focused on expanding RPE cells in culture may be able to address this problem [103–105]. The clinical use of human fetal-derived retinal tissue is arguably controversial, and the supply is very limited and insufficient to treat millions of people suffering from blindness. Phase II clinical trial done in AMD (NCT00346060) and RP (NCT00345917) patients (Table 1) showed successful transplantation of fetal retinal tissue along with RPE in the subretinal space. This treatment improved visual function in some patients without causing immune rejection [106].

### Human Embryonic Stem Cells

Human embryonic stem cells (hESCs) are derived from the inner cell mass of a blastocyst (an early-stage, day 5 cultured preimplantation embryo), maintain karyotypic stability in culture [107–110], and are considered a limitless source of pluripotent stem cells for cell therapies [9, 111]. Human ESCs are a promising source for cell therapies to treat blindness [112–115]. Substantial progress has been made over the last two decades in derivation of monolayers of RPE cells from hESCs either spontaneously or via directed differentiation [72, 115–120], which are highly similar to primary human RPE cells [121]. The stem cell-derived RPE appear very similar to human fetal RPE, as they form hexagonal pigmented monolayers and express markers specific to RPE (BEST1, ZO-1, MITF, RPE65) [72, 115, 122, 123]. These RPE cells mature as adult human RPE cells and have the ability to phagocytose photoreceptor outer segments in vitro and in vivo [118, 122], which is a critically important RPE function to enable rod outer segment renewal in the visual phototransduction cycle [124]. Transplantation of hESC-derived RPE as a single cell suspension showed improvement in visual performance in the Royal College of Surgeons (RCS) rats [72, 125]. Importantly, hESC-RPE safety study results demonstrated that transplanted hESC-RPE cells survived in the subretinal space of RCS rat for more than 200 days without causing tumorigenesis [126]. Human ESC-derived RPE cells have been recently tested in human clinical trials of Stargardt’s macular dystrophy (NCT01345006) and Geographic Atrophy (GA, dry AMD patients, NCT01344993) [114, 127, 128]. Initial results showed no evidence of adverse cell proliferation and immune rejection. Improvement of visual function was reported in 10 treated eyes after 22 months following transplantation [127]. Similarly, in Asian clinical trials, transplantation of hESCs derived-RPE cells in four Asian patients (two with dry AMD and two with Stargardt’s macular dystrophy) revealed no evidence of serious safety issue and tumorigenicity. In three patients, visual acuity improved 9–19 letters during 12 months of the follow up study [129]. Interestingly, while positive impact of RPE grafting may be explained in dry AMD cases (where the recipient RPE degenerates, causing secondary photoreceptor degeneration), understanding the modest improvement of vision after RPE transplantation in retina affected by SD needs further investigation. Stargardt’s macular dystrophy (*fundus flavimaculatu*s, SMD, or juvenile macular dystrophy) is mostly caused by mutations in ABCR (ABCA4) [130] or less often, ELOV4 genes, both causing photoreceptor (not RPE) cell death. Therefore, the positive impact of RPE transplantation into AMD patients is possibly due to trophic support by RPE of the patient’s photoreceptors, rather than cell replacement effect. Currently, Cell Cure Neurosciences, Ltd (a fully owned subsidiary of BioTime, Inc.) is doing a safety and efficacy phase I/II clinical trial of dry AMD (NCT02286089) at several locations in Israel and USA using RPE cells (OpRegen® product), derived from cGMP-grade hESCs [131] and tested in the Royal College of Surgeons (RCS) rats [73].

**Fig. 2:**
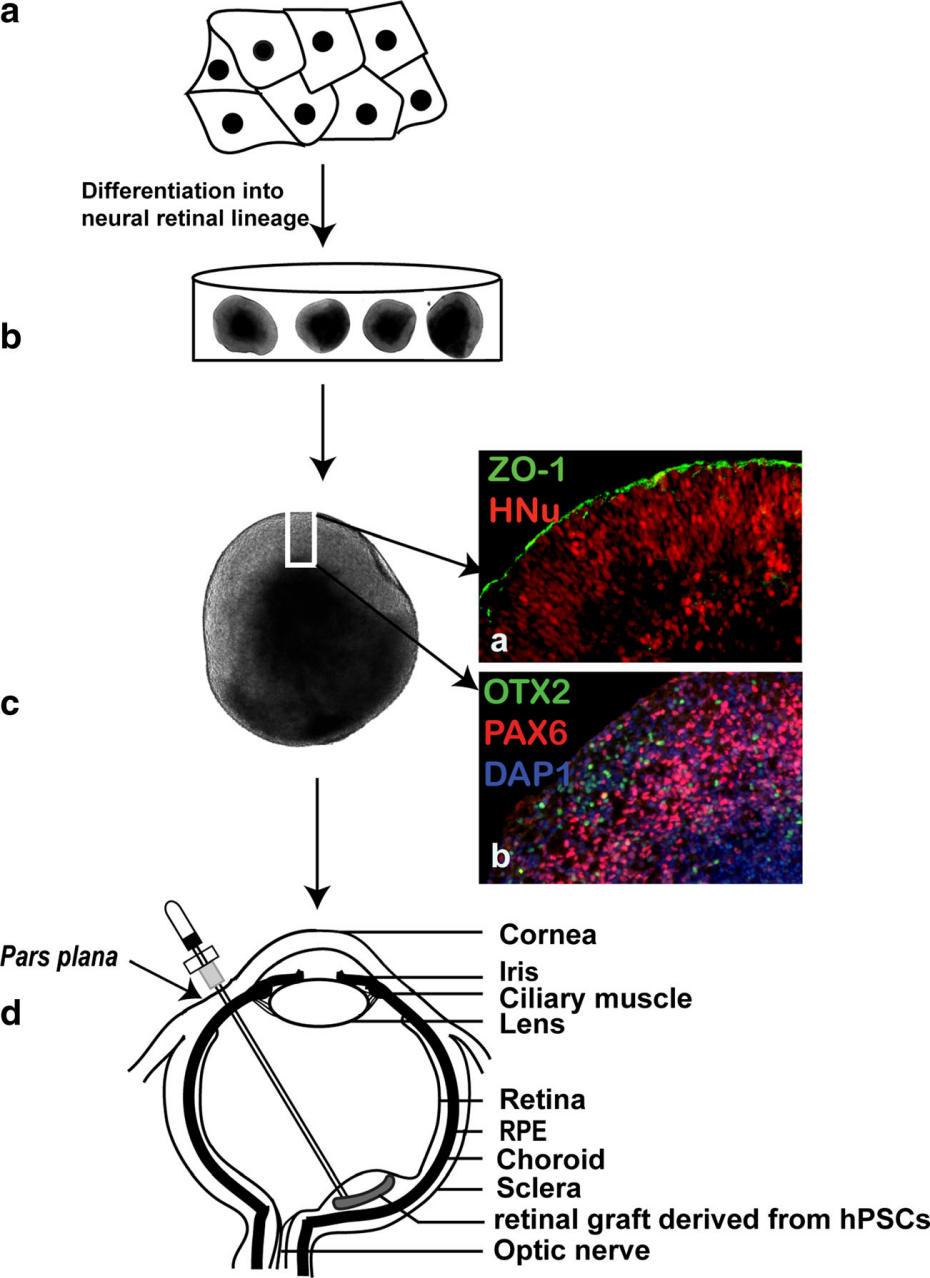
Schematic diagram showing transvitreal grafting of retinal tissue derived from hPSCs in subretinal space. **(a**) human pluripotent stem cell differentiated into retinal lineage. **(b**) Three dimensional retinal organoids growing in a dish. **(c**) Phase contrast image of retinal organoids showing different layers differentiating within retinal organoid. (a) hESCs derived retinal organoid coimmunostained for RPE marker ZO-1 and human nuclei marker HNu. (b) Retinal organoid coimmunostained for multipotential retinal progenitor markers OTX2 and PAX6. DAPI stained nuclei. **d**. A piece of retinal tissue derived from hESCs is transplanted transvitreally into subretinal space

Human embryonic stem cells can be directed to retinal cell fate by inhibiting WNT, BMP (and NODAL) signaling using potent morphogenic proteins (Dickkopf-related protein 1 and Noggin), as well as an array of small molecules, related morphogenic proteins and their combinations [113, 132]. Further culturing cells with insulin-like growth factor 1 (IGF-1) and basic fibroblast growth factor (bFGF) promotes the survival of RPCs, induces the expression of eye field transcription factors (EFTFs) and more mature retinal immunophenotype markers (Fig. 2). Subretinal injection of hESC-derived RPCs in *Crx* ^−/−^ animal model of retinal dystrophy demonstrated partial vision rescue (restoration of ERG *b*-wave) and evidence of synaptic connectivity with recipient bipolar cells [112], indicating cell replacement rather than neuroprotective mechanism behind vision improvement. Chao et al. injected hESC-derived RPCs into the subretinal space of non-human primate (*S. sciureus*) and reported cell survival for at least 3 months without immunosuppression. While functional integration of donor cells was not achieved, hESC-RPCs demonstrated maturation and extended axonal projections into the host inner retina and optic nerve [133].

Seminal work done by Sasai and colleagues have established a protocol for differentiating hESC to self-organizing 3D retinal organoids containing distinct retinal cell types and exhibiting stratification of neuronal layers [5, 134, 135]. Retinal organoids are very similar to developing human fetal retina and display the presence of proliferating/migrating RPCs, RPE, photoreceptors, second order neurons (INL) and RGCs. Later, several studies reported derivation of similar mini retina-like structures from hESC under different culturing condition [19, 120, 135–140]. Recently, Shirai et al. transplanted hESC-derived 3D-retinal tissue into monkey model of retinal degeneration. Grafted hESC-retinal tissue differentiated to different retinal cell types including rod and cone photoreceptors and formed synaptic connectivity with the host retina [20]. To date, there is no report of hESC-derived retinal tissue transplantation in subretinal space of human patients.

### Induced Pluripotent Stem Cells

Extensive cutting edge work, done by Takahashi and Yamanaka in 2006, has lead to the identification of four key reprogramming transcription factors: OCT3/4, SOX2, C-MYC and KLF4 that can reprogram differentiated somatic cells to ESC-like cells [141]. Their results were succesfully confirmed by several groups using different combinations of transcription factors, such as OCT4, SOX2, NANOG and LIN28 to avoid the use of MYC, a known proto-oncogene [11]. After reprogramming, cells acquire pluripotency, where they can self-renew and differentiate to any type of cell in the body. Protocols to differentiate human induced pluripotent stem cell (iPSC) line to RPE and retinal progenitor cell were successfully established by multiple groups [116, 123, 142–145]. The RPE cells derived from iPSCs form a monolayer of pigmented cells that shows the feature of polarity, form tight junctions and have a tendency to phagocytose photoreceptor outer segments. Transplantation of iPSCs-derived retinal cells into animal models of retinal degenerative diseases showed efficacy in preserving visual function [146, 147]. Recent reports demonstrated that iPSCs can generate optic vesicle- and optic cup-like 3D structures and produce retinal progenitors that differentiate into RPE, photoreceptors, inner nuclear layer (INL) neurons and ganglion cells (RGCs). Culturing iPSC-derived retinal spheres in suspension for up to 6 months revealed the ability of retinal organoids to form cell layers, including PRs with outer disk-like protrusions and photosensitivity [138, 139], which are challenging to purify in two-dimensional culture. Transplantation of dissociated and purified iPSCs-derived PR progenitors into the subretinal space of wild type mice showed that grafted photoreceptors migrated and settled in ONL of the host retina and have protein expression very similar to that in wild type mouse photoreceptors [144]. Barnea-Cramer et al. transplanted hESC/iPSC-derived photoreceptor progenitor cells in 10–12 week old *rd1* retina and found some light response by behavioral tests. However, transplanted photoreceptors did not develop mature morphology with outer segments as the animals were terminated at three weeks after surgery [148].

Induced pluripotent stem cells discovered by Yamanaka and Takahashi provided a reproducible method of deriving autologous hPSCs for every patient [141]. However, when all the costs and time for (i) generating single batch autologous cGMP-grade iPSCs and (ii) quality assurance testing (including identity, potency, sterility, karyotyping and whole-genome sequencing/sequence analysis) are taken into account, it is unlikely that autologous iPSC technology would be routinely used for cell therapies. A recent finding of an oncogene mutation in a genome of an iPSC cell line produced for generating a RPE patch for autologous RPE replacement in an AMD patient [149] reinforces the concern that using iPSC technology for each and every patient would not best cost effective and therefore could limit patient access. In addition, reports about some retained epigenetic memory in iPSCs [150] raise the possibility that the (retinal organoid) differentiation protocols may not produce consistent results when applied to iPSCs from different patients. Prolonged in vitro culture and genetic manipulations may change the genome of iPSCs, including histocompatibility [151], potentially causing graft rejection. Collectively, given the immune privileged status of the subretinal space, low-passage histocompatible cGMP-grade hESCs from stem cell banks with a verified genome and epigenetic signature may be a far more feasible alternative for hPSC-retinal tissue clinical applications, from both regulatory (Quality Assurance/Quality Control [QA/QC]), biological (reproducibility) and commercialization (manufacturing costs) perspective.

## Challenges in Advancing hPSC-Derived Retinal Tissue Technology to Clinic

While we remain optimistic to find ways to repair retina by injecting a suspension of multipotential retinal progenitors or photoreceptor progenitors, there is no definitive evidence that these technologies will robustly work in clinic for blind patients whose retina already degenerated beyond repair. A small number of photoreceptor progenitors indeed is capable of crossing the outer limiting membrane and integrating into retina [112, 144, 152–154], although the reported numbers of photoreceptor are unlikely to restore useful vision. Photoreceptors need retinal architecture to find guidance cues [155] to integrate, and contact with apical RPE to survive. Although some do integrate indeed, the majority do not, leaving a donor cell mass in the subretinal space [98, 113, 153, 156, 157]. Moreover, the neurodegenerative environment of *rd* retina was shown to cause death of transplanted photoreceptors [158, 159], when the fate of integrated cells is tracked for several months. This matches many [160–164] yet not all [165–167] reports in brain, where the neurodegenerative milieu of the brain caused transplanted neurons to acquire a disease phenotype. Therefore, while retinal tissue derived from hPSCs provides us an opportunity to move vision restoration therapies forward, major challenges lie ahead in the quest to optimize hPSCs based retinal therapy.

### Tumorigenicity

Stem cells have a striking resemblance to cancer cells. Uncontrolled cell division of hESC and iPSC often leads to tumor growth after transplantation in vivo [168]. When hPSCs are injected into immunodeficient mice, they form teratomas - nonagressive tumors containing differentiated cells representing all three embryonic germ layers [12, 169–172]. In immunocompetent MHC-mismatched mice teratoma formation is usually suppressed, unless PSCs aquire a more agressive and invasive teratocarcinoma-forming activity [168]. Growth of such teratomas is influenced by the graft site (the niche) [173]. Retinal organoids are the product of advanced differentiation of hPSCs to an anterior neuroectoderm > eye field > optic vesicle > optic cup-like structure, where many cells already acquired their final differentiated state and established rudimentary lamination within the hPSC-retinal tissue. When transplanted into subretinal space, hPSC-derived retinal cells and tissue undergo further differentiation and maturation induced by the subretinal niche environment [20, 21, 174, 175] regardless whether the recipient retina is degenerating or not. Hambright et al. earlier demonstrated that hESC-derived retinal progenitors acquire photoreceptor cell fate in subretinal, but not epiretinal space of adult mice without retinal degeneration [113]. Singh et al. found that even in advanced stages of degeneration (when all photoreceptors are gone), rod photoreceptor progenitors grafted in subretinal space receive permissive cues for maturation [157]. Collectively, the danger of tumorigenesis caused by PSC-derived retinal tissue grafted into subretinal space seems to be a very remote possibility unless (i) the transplanted tissue is surrounded by immature cells, and (ii) is derived from a PSC line which has not been rigorously checked for chromosomal abnormalities/genomic changes. Assuming that hPSC-derived retinal tissue would be generated from rigorously tested low passage hPSC lines from a stem cell bank, previously tested in animal subretinal space, the chances of tumorigenesis are very remote. In addition, the selection of a 3D retinal organoid growing as attached or floating aggregate provides an additional way of positive selection against immature cells of neural origin, which may be a by-product of organoid differentiation. Clinical trials completed on subretinal transplantation of hESC-derived RPE cell suspension in AMD and Stargardt’s disease patient found no report of adverse cell proliferation [114, 127]. Schwartz et al. summarized a 4-year follow up study in AMD patients, who received hESC-derived RPE grafts [128]. No tumors were reported in this study. Recently, iPSC-derived RPE cells were transplanted to a patient with neovascular AMD and no serious events were observed at two years of follow up [176]. Later on RIKEN research institute in Japan halted this clinical trial due to identification of potentially oncogenic mutation in one of the 2 patients. This rigorous work further highlighted the challenges of working with individual iPSCs and suggested using banked cGMP hESCs to avoid encountering similar problems in the future.

### Immunogenicity of hPSC-Derived Retinal Grafts and Prospects for Circumvention

Human pluripotent stem cells possess immune-privileged properties and their terminally differentiated derivatives were reported to be less susceptible to immune rejection than adult cells [177–180]. Human embryonic stem cells express low levels of human leukocyte antigen (HLA) class I molecules in the resting state, with a limited induction during differentiation and do not express HLA class II molecules [181]. Similar results have been reported for hiPSC [182]. Allogeneic transplants made from hESC-derived terminally differentiated derivatives caused an immune response and were rejected [179]. This process was explained by various mechanisms. First, activation of allogenic natural killer cells eliminate hESCs [183, 184]. Second, hESC express low level of HLA class I molecules and also OCT4 that can indirectly activate T cells through antigen presenting cells [177, 185]. Third, after transplantation hESCs undergo differentiation into various cell types that express HLA molecules, leading to robust T cell-dependent allogenic rejection [186, 187].

Induced PSC technology raised the hope that patient-specific iPSCs will be autologous and thus will not be rejected by patient’s immune system [188]. However, recent studies have demonstrated increased genomic instability, epigenetic abnormality and immunogenicity of iPSCs raising safety concern of iPSCs based cell therapy [189–192]. A comprehensive summary of hPSC immunogenicity and hPSC-derivatives was provided by de Almeida et al. [193], Zhao et al. [186] and Boyd et al. [151]. Boyd et al. raised the question whether patient-specific iPSCs would necessarily be histocompatible to the patient [151]. It appears that genome of iPSCs may change during genetic correction of a disease-inducing mutation or prolonged in vitro culture, which is sufficient to trigger immune reaction and graft rejection [151]. Studies of iPSC immunogenicity in mice showed that epigenetic differences between ESCs and iPSCs may lead to the upregulation of genes that induce a T cell response, resulting in the rejection of transplanted cells and tissues in syngenic recipients in contrast to ESCs of the same origin [186, 187]. Thus, immunosupression and/or hematopoietic chimerism may be needed to proceed with grafting hPSC-derived cells or tissues [151]. The subretinal space, where the hPSC-3D retinal tissue is intended to be grafted, is surrounded by the photoreceptor layer on one side, and the RPE layer on the other side, both avascular, which may explain the given “full immune privilege” status. Hambright et al. reported the survival and differentiation of completely xenogenic (hPSC-derived retinal progenitors) grafts in adult mouse subretinal space for up to 3 months without any immunosuppression [113]. Others demonstrated that long term survival of transplanted neural retinal tissue can be achieved in human patients [194–196] and monkeys [197] without immunosuppression. On the contrary, Zhu et al. transplanted hESC-derived retinal cells into IL2rγ-deficient mouse retina and reported improved integration of donor cell into the host photoreceptor layer [198]. Their data indicate that immunosuppressed retinal environment provides long term survival of donor cells. A number of studies also proposed using local immunosuppression because locally (intravitreal) immunosuppressed transplants survived in the subretinal space longer than those without intravitreal immunosuppression in their experiments [199]. This report noted that immunosuppression can be effective at a local level and with a relatively low concentration of cyclosporine. Others used a low concentration of systemic cyclosporine and noted that human CNS stem cells grafted into the subretinal space are “not significantly immunogenic” [197].

In AMD, immune privileged status of ocular space is breached due to the breakdown of the BRB, potentially caused by changes in BrM/RPE leading to inflammation, activation of microglia/macrophage/ complement factor protein present in drusen and surge in T cell mediated immune system [56–58]. Therefore, in the first hESC-RPE clinical trial of AMD, patients received systemic immunosuppression one week prior to grafting, and for the next twelve weeks after grafting [114, 127] to account for a potential breach of the BRB during surgery and to prevent the immune system from destroying the nonautologous RPE grafts. Once the BrM heals and BRB is repaired, the assumption is that there would be no need for life-long immunosuppression, which is associated with cancer [200]. An alternative approach to obviate the immune rejection caused by hPSCs-derived retinal tissue is to use PSCs from a stem cell bank and select PSCs with at least partial immunological match to a patient [201]. Pluripotent stem cell bank consisting of 200–300 lines provides reasonable expectation of finding a partial immunological match to a prospective patient [201–203]. Recently Cellular Dynamics International (CDI) made a cGMP grade HLA superdonor master cell bank using proprietary nonintegrating episomal vector technology [204] to generate iPSCs from such HLA superdonors. On the contrary, RIKEN is using an iPSC bank for AMD therapy, where cells are collected from healthy donors (allogenic) instead of using patient-derived iPSCs (autologous).

### Mode of Delivery of Cells and hPSC-Derived Retinal Tissue in Subretinal Space: Trans-Vitreal and Trans-Scleral

One of the major hurdles in advancing hPSC-derived retinal tissue to clinic is optimizing the surgical technique for delivering a piece of retinal tissue in the subretinal space. Earlier studies have shown three routes for delivering suspension of RPE cells in the subretinal space of rodent models: “Open sky”, transvitreal (via *pars plana*) and transscleral-transchoroidal-Bruch’s membrane. In the “Open Sky” approach the cornea is removed, and the globe is exposed during the surgical procedure [205]. A major disadvantage this technique is the risk of causing trauma to the retina, vitreous and anterior chamber. The trans-vitreal approach involves doing a vitrectomy first, followed by inserting a microcapillary glass pipette through the *pars plana*, avoiding the lens, through the vitreous space and into the retina. Limitations of this technique include potential damage to the retinal layers, also the possibility of triggering gliosis and causing proliferative vitreoretinopathy (PVR) [206]. The trans-scleral-trans (supra) choroidal-BrM approach involves inserting a polished glass microcapillary [207–209] or special catheter through the sclera, choroid and BrM and into the subretinal space without penetrating the retina. The catheter was originally developed for glaucoma treatments [210] and subretinal delivery of drugs [211–214] and later adapted for cell transplantation (NCT01226628 by Janssen Research & Development, LLC, iTrack catheter) [215]. Limitations of this technique includes technical challenges, as well as potential rupture of BrM, causing subretinal hemorrhage due to penetration of choriocapillaris and eventually causing inflammation and immune response. The big advantage of this approach is lack of retinal injury (and subsequent gliosis and PVR) and no damage to the lens [208]. In large eyes, the transvitreal surgical approach is considerably easier. Wongpichedchai et al., compared external (transscleral-transchoroidal-BrM) vs internal (transvitreal) approaches in rabbits and found similar results with both the approaches [216]. Recently, transvitreal approach has been the only approach used for the delivery of donor cells in clinical trials [114, 127, 128] and in preclinical studies focused on 3D retinal tissue grafting [20, 21, 217].

### Aberrant Synaptogenesis and Retinal Gliosis due to Retinal Degeneration Impedes Transplantation

Retinal repair by RPE transplantation is moving forward quickly and generating hope supported by evidence from clinical trials [114, 127] and research data on animals [72, 218, 219]. RPE is a single layer of supporting cells and is not required to establish synaptic connectivity. However, in the case of neural retina, the task of functional replacement of retinal neurons is substantially more challenging. The new cells need to migrate to specific neuroanatomical locations in the retinal layer and re-establish specific synaptic connectivity in the synaptic architecture of the host retina, which is degenerating. Synaptic remodeling of neural circuits during advancing retinal degeneration further complicates this task [220, 221]. Aberrant synapsing between hESC-3D retinal tissue and synaptic circuitry of recipient degenerating retina may, theoretically, worsen and/or distort remaining vision. Retinal degeneration induces synaptic remodeling in RP animal models. In early phase 2, stressed photoreceptors develop abnormal sprouting of neurites, which often reach the inner plexiform and ganglion cell layers, “overshooting” their synaptic targets [220, 222]. Such rewired circuits distort retinal processing and in late stages of retinal degeneration may not support vision [223]. According to Seiler and Aramant, INL neurons of the grafted retina need to synapse on RGCs of the recipient neurons in order to restore the circuitry and activate *superior colliculus* [217].

Extensive gliosis by Müller glial cells during retinal degeneration leads to the formation of a glial scar. Gliosis progressively increases with retinal degeneration progression, yet in different retinal degenerative diseases the dynamics may be different, which has a direct impact on the neurite extensions as well as the ability of grafted cells to migrate into the host retina [224]. Retinas of mice deficient in glial scar formation (GFAP ^−/−^, Vim ^−/−^) were reported to be more permissive for both cell migration and axonal elongation [225], highlighting the major negative impact of reactive gliosis on the ability of transplanted 3D retinal tissue to elongate axons and synaptically connect with recipient retinal tissue. Current solutions to attenuate the glial response after transplanting a 3D retinal tissue are limited. Some immediate solutions are likely to be focused on improving surgical delivery [226], as well as optimizing the size of the graft [227] to minimize reactive gliosis after grafting.

### Limitation Associated with hPSCs-Derived Retinal Tissue Size and Composition

Several key technical and biological problems associated with lab-grown retinal tissue design need to be addressed to enable this technology to work in clinics. The size of the human fetal retinal tissue successfully used for clinical trials was 2-5mm^2^ (average size 3.8mm^2^) [106]. Given that one degree of visual angle is equal to 288 *µ*m (0.288 mm) of the retina without correction for shrinkage, [228, 229] 3.8mm^2^ slice of retina may support 13.2 degrees of visual field angle, which is a significant step forward in retinal degenerative therapies. However, the size of 3D retinal tissue derived from hPSCs is much smaller (approximately 0.5 mm length or less), and the photoreceptor density is lower than that in human fetal retina. Therefore, a single retinal organoid piece may not be able to produce measurable beneficial changes in visual perception in a large eye such as human, though it may do so in a small rodent eye. Retinal organoids growing in stagnant conditions in a dish are devoid of nutrients and oxygen. Improving transport of nutrient and oxygen by growing retinal organoid in higher oxygen conditions [230] and/or in rotating-wall vessel (RWV) bioreactors [231] are the right steps forward for improving retinal organoid technology. Another way to improve retinal organoid design is increasing the number of photoreceptors in hPSC-retinal tissue, for example by using agonists or inhibitors of known developmental pathways active in retina, such as Notch [139, 232] and/or other signaling molecules [233]. An alternative feasible approach is designing a retinal patch consisting of a number of flat pieces of hESC-3D retinal tissue stitched together. Such “bioprosthetic” retina may bring a considerable number of photoreceptors into the subretinal space of blind patients, enabling at least partial restoration of light sensitivity, for as long as the graft can synapse on recipient retinal cells.

### Manipulating Recipient Retina’s Extracellular Matrix (ECM) and Outer Limiting Membrane (OLM), to Facilitate Functional Integration of Subretinal Grafts

The disease environment of the recipient retina clearly has a significant yet not fully understood impact on structural and functional integration of retinal grafts, though it varies in different retinal degenerative conditions [158, 224, 234], further confounding the conclusions and comparing the data reported by different groups. Because almost all work on cell replacement in retina has been done using dissociated cells (rather than intact retinal tissue grafts), major emphasis of retinal cell replacement work has been focused on facilitating cell migration into the recipient retina. There is no need for cell migration when a 3D retinal tissue graft is deposited into the subretinal space. However, axonal migration is needed to enable synaptic integration of the donor retinal tissue into the synaptic circuitry of the recipient retina with retinal degeneration. Here we briefly list the major known biological obstacles interfering with structural and functional integration of subretinal grafts, using the experience developed in transplanting suspension of dissociated retinal cells, and discuss their likely impact on connectivity between donor retinal tissue and the recipient retina.

*Extracellular matrix* is composed of proteins and sugar molecules, which may hinder or promote incorporation of newly transplanted cells into recipient retina. Their impact on synaptic integration of 3D retinal tissue grafts is likely minimal. Among the ECM components the roles of chondroitin sulfate proteoglycan (CSP) and matrix metalloproteinase-2 (MMP-2) were repeatedly investigated. CNS (brain, spinal cord and retinal) extracellular milieu is rich in proteoglycans. The chondroitin sulfate proteoglycan was reported to be inhibitory for migration of brain and retinal progenitor cells [235] and therefore an obstacle to CNS repair [236]. Localized degradation of CSP with bacterial enzyme chondroitinase ABC promotes functional recovery after spinal cord damage [237, 238] as well as migration and integration of transplanted retinal cells in subretinal space [239, 240]. However, CSP expression was noted to be high in the embryonic CNS (which is the most permissive to synaptic integration of transplanted brain and retinal progenitors). Moreover, CSP was reported to direct axonal outgrowth [241], making it a plausible yet unlikely obstacle for functional integration of 3D retinal tissue grafts. Manipulation of MMP-2 (gelatinase-A) was suggested to have a role in improving cell migration [242], which is not needed for synaptic integration of 3D retinal tissue graft. Though retinal ECM may be inhibitory for outgrowth of mature retinal axons, the axons from young (embryonic) retinal tissue grafted into the subretinal space are expected to be heavily polysialylated (post-translational modification by the addition of polysialic acid [PSA] to the fifth Ig domain of neural cell adhesion molecule, NCAM), enabling easier outgrowth. PSA-NCAM is high in young axons but disappears in mature axons, which was reported to be associated with axonal myelination (myelination increases, while polysialylation decreases with axonal/neuronal maturation) [243]. Among the studied roles of PSA-NCAM on young axons are axonal fasciculation (axonal outgrowth and target finding) [244, 245]. PSA-NCAM increases in RGC axons after injury, and positively correlates with repair/remodeling [246].

During brain development, polysialylated (polySia) neural cell adhesion molecules (polySia–NCAMs) modulate cell–cell adhesive interactions involved in synaptogenesis [247]. In summary, the presence of PSA-NCAM on young axons outgrowing from young 3D retinal tissue may help minimize the inhibitory influence of ECM in the recipient retinal degenerative retina and promote axonal outgrowth and synaptic connectivity with the recipient retina.

*Outer limiting membrane (OLM)* is the strong natural anatomical barrier between the subretinal space and the ONL, preventing migration of cells from the subretinal space into the recipient retina in the normal healthy retina [113, 207]. Targeted disruption of OLM improved integration of transplanted photoreceptors from subretinal grafts into the recipient retina [248, 249]. OLM becomes structurally compromised (porous) in some retinal degenerative conditions more (e.g., *Crb-1* mutation, *rd8* [250]) than in the other. This improves not only cell integration but also contributes to variable outcomes after cell transplantation into *rd* models [153, 251]. On the contrary, in healthy retina transplanted retinal progenitor cells do not migrate from subretinal space to the recipient retina [113, 207]. OLM is located at the base of rods and cones (between the photoreceptor nucleus and the inner segment) and is comprised the adherens junctions (*zonula adherens*)/desmosomes between the end feet of Müller glia and photoreceptor inner segments. OLM is likely not a barrier in most advanced retinal degenerative conditions (which are precisely the targets for 3D retinal tissue grafting therapies, where all/most host photoreceptors are degenerated), as it becomes porous due to retinal degeneration and structural changes in retina and thus more permissive for axonal migration. Whether OLM represents a structural barrier for 3D retinal tissue grafts before the onset of advanced retinal degeneration remains to be determined. The importance of OLM for structural preservation of retina as well as its implicated role in maintaining retinal barrier [252] suggest that it may indeed a physical barrier to axonal connectivity between the retinal tissue grafts and the synaptic circuitry of the host retina.

### Exchange of Cytoplasmic Material as a New Concept in Retinal Cell Replacement Therapy

Earlier studies have shown that transplantation of healthy photoreceptor precursor cells in diseased retina partially restores vision. This phenomenon was ascribed to donor cell migration and integration to the host cell [94, 112, 153, 156, 253]. Recently this concept has been confronted by multiple reports [254–258], all providing strong evidence that the donor photoreceptors in subretinal space undergo material transfer (cytoplasmic exchange) with the host photoreceptors instead of donor cell integration to the host photoreceptor layer. Pearson et al. demonstrated that material transfer does not involve donor host nuclear or cell–cell fusion, or the uptake of free protein or nucleic acid from the extracellular environment. Instead, RNA and or protein are exchanged between the donor and host cells [258]. The actual mechanism behind the cytoplasmic fusion between donor cells and the host is speculated to be happening through tunneling nanotube and/ or vesicular transport such as exosomes [255, 259, 260]. Interestingly, a recent study by Waldron et al., demonstrated that transplantation of cone photoreceptors in murine model of retinal degeneration results in both donor cell integration and cytoplasmic fusion and the whole process is dependent on the etiology of disease and host retinal environment [261]. The conclusion reported by Waldron et al. could be foreseen when a developing neural graft is placed in the proximity to the mature and degenerating neural (retinal) tissue. One may expect some axonal outgrowth and human puncta in the recipient retina, in addition to some exchange of cytoplasmic material due to some cell death and release of cytoplasmic proteins in the extracellular space. In addition, one may expect some transsynaptic exchange of proteins [262, 263], in the case/when chimeric graft/recipient synapses mature [227].

### Graft Survival and Host Integration: Using Biomaterials

Therapeutic cell transplantation studies in retina suffered from a significant drawback due to cell aggregation, cell death and lack of integration of grafted cells with host retina [264]. In contrast, fetal retinal tissue transplanted as a sheet [95] or even as aggregates [97] survived well in subretinal space, because continuous adherence to a supportive RPE sheet of a recipient [95], and potentially the presence of paracrine support from neighboring retinal cells in grafted tissue, protected them from anchorage dependent cell death known as anoikis [265]. Interestingly, 3D retinal organoids growing under in vitro condition show a certain degree of variability in cellular composition and developmental dynamics, which makes them different from native fetal retinal tissue. Retinal organoids grown in vitro show a high level of heterogeneity within the same cell lines, as well as variability between the cell lines. The number of retinal cell types present in retinal organoids is different from that present in native fetal retinal tissue. This suggests that microenvironmental cues like chemical, biophysical and/or cellular are either lacking or imbalanced in space and time during tissue growth in 3D culture [266]. We believe that combining optimized biomaterials with stem cell derived retinal organoids will eventually enable faithful recapitulation of in vivo fetal retina development in a dish. Biomaterials are natural or synthetic matrix molecules that replicates innate extracellular matrix in various tissue. Biomaterials are used for enabling the stem cells to survive, differentiate and also to create artificial tissues [267, 268]. The concept of applying biomaterials for engineering (and /or supporting) growing hPSC-retinal tissue is not yet sufficiently well developed to be applied in patients. This is in contrast to a wealth of published research on biomaterials for corneal engineering [269], demonstrating a principal difference in logistics when approaching engineering of mammalian retina, and/or reflects the magnitude of the challenge involved in printing neural tissue. There are generally two main purposes in using biomaterials to support tissue repair. First the biomaterials provide structural elements or cues to the graft. Second, they facilitate grafts implantation in a specific anatomical site by redesigning a niche. Attempts were made to deposit layers of retinal progenitors onto biomaterials-guided matrix to recreate retinal neuroanatomy [270]. It is likely that biomaterials will be used to both enhance hPSC-3D retinal tissue during growth and/or grafting [5]) as well as to assemble retina de novo. Physical parameters of human retina need to be taken into account when engineering human retina either by inducing hPSCs to form retinal organoids or by any other means. The thickness of mammalian retina is about 0.5 millimeters (mm), with the thinnest area at the foveal floor (0.15–0.20 mm) and thickest area at the foveal rim (0.23–0.32 mm) [228, 271]. For any retinal replacement strategy utilizing engineered or differentiated retinal tissue, the thickness of such retina must be comparable to the thickness of the recipient retina to avoid causing mechanical distortions of recipient tissue [272]. This will preserve viability, mechanotransduction properties, physical connectivity and ultimately promote engraftment (including vascularization) and restoration of visual function. Transplanting any graft, which will cause the host retina to be locally pushed toward the lens may cause a strong defocusing (hypermetropia-like) and distortion of the retinal images in the rescued area and as a consequence, mechanical distortion of cellular membranes and axons may lead to Wallerian degeneration [273, 274]. The biomaterial used for retinal grafting is viewed as porous, biodegradable, with a correct Young’s modulus (a measure of the stiffness of a solid material), and thinner than 50 *µ*m [275]. Among the polymers, which match these criteria are poly(lactic-co-glycolic acid) (PLGA), poly(lactic acid (PLLA), poly(glycerol-sebacate) (PGS), and poly(caprolactone) (PCL) [269]. PLLA-PLGA combination was used for engineering a scaffold for RPE transplantation [276]. However, entirely new attributes may be required for biomaterials used to support hESC-3D retinal tissue in subretinal grafts (rather than a RPE monolayer on a PLGA/PLLA scaffold), as such biomaterials may help to keep 3D retinal tissue from forming tubular structures in subretinal grafts [20, 21]. We should likely expect a rapid development of novel biomaterials in the future promoting 3D tissue adhesion, engraftment, survival, lamination, differentiation, synaptogenesis, axonogenesis and connectivity with the host retina to restore vision.

Recent advances in decellularized scaffold techniques are expected to preserve tissue architecture and chemistry [277]. This technology was used to recreate a hepatic niche from decellularized 3D liver organoids [278] seeded with liver cells and vascular cells. Decellularized retina can be generated from cadaver eyes. However, retinal tissue requires not only structure but precise synaptic connectivity between retinal layers to function properly. In addition, dissociation of adult retinal neurons requires disruption of retinal structure, and usually causes rapid neuronal cell death, leaving Müller glia as the only viable cell type in a dish. Decellularized retina can be seeded with multipotential retinal progenitors [279]; however, it is very unlikely that the same developmental cues guiding migration of retinal progenitors to retinal layers in the embryonic retina are preserved in decellularized cadaver retina to recreate a 3D retinal neuroanatomy, needed for capturing and propagation of visual information. There are no reports confirming this at the moment. Neuroprosthetic devices such as ARGUS-II (an artificial retina, 64 pixels, manufactured by the Second Sight), or Alpha EMS (1,500 microphotodiode-amplifier pixels, manufactured by Retina Implant AG) already enabled patients to regain some independence, with new versions of neuroprosthetic devices on the way (e.g., wireless photovoltaic subretinal prosthesis from Pixium Vision [280]). Retinal tissue from hPSC may be a timely addition to development of these technologies aimed at restoring vision by designing and transplanting sensors into the ocular space and reconnecting them to remaining neural circuitry of blind patients.

Collectively, our current understanding and knowledge of dynamically changing extracellular matrix within and around the rapidly growing neural tissue (and retina in particular) is very incomplete, and requires the concerted work of teams of engineers and developmental neurobiologists and stem cell researchers to succeed in attempts to reengineer functional 3D retinal tissue.

## Commercialization of hPSCs-Derived Retinal Tissue Technologies

Stem cell derived retinal organoid technology must overcome pre-market and post-market commercialization challenges. A major premarket challenge is finding a stable funding source that can fund both preclinical (in vivo) and clinical work (clinical trial) while maintaining compliance with regulatory processes. Post market problems include (i) finding the right reimbursement mechanisms, (ii) working with providers to convince them to change their treatment approach, and (iii) working with payers to develop the codes to cover the treatment procedures. If a medical procedure has no insurance reimbursement codes then stem cell procedure cannot be provided in a hospital and cannot be covered by insurance [281]. Challenges common to both pre- and post-market phase include scaling up production, distributing cell therapies, and reducing the costs of production.

### Premarket Challenges: Bridging the “Valley of Death”

Because vision loss is so devastating, the importance and urgency of this technology is immediately understood by donors, foundations, patients, FDA, and providers alike. The stem cell-based Regen Med technology provides therapeutic potential in indications where pharmacological and surgical treatment approaches are ineffective or simply not applicable. This creates major medical and commercial opportunities for new clinical trials in vision restoration. Stem cell-mediated treatment of blindness received FDA’s Regenerative Medicine Advanced Therapy (RMAT) designation in 2016 (as part of Congress-approved twenty-first Century Cures Act signed into law on Dec.13, 2016), allowing expedited review and approval of promising stem cell treatments of life-threatening conditions. An eligible stem cell therapy is expected to demonstrate preliminary clinical data having the potential to address an urgent unmet need. Companies working on vision restoration are already taking full advantage of this opportunity. RMAT allows new promising treatments to be tested in clinical trials faster, enabling biotech companies to recover the high R&D cost of developing new stem cell treatments more quickly. This is expected to help overcome major translational challenges of moving stem cell therapies from preclinical phase to clinical trials [149, 282–285], improve the efficacy [286] and keep business models viable to enable further translational work. Retinitis Pigmentosa is an orphan class disease with the potential for a fast-track FDA-regulated path to clinical trials, further contributing to the acceleration of translating bioprosthetic retina from preclinical testing in animals to human clinical trials. Although producing positive preclinical proof-of-concept data certainly opens the door to potential corporate partnerships, moving from the preclinical stage to clinical proof-of-concept requires more capital than preclinical validation in animals. The gap between these two periods is often referred to as the “valley of death” because it is when many novel therapeutics fail to secure funding for further development [287, 288]. Recognizing this funding challenge and adequately preparing for it by raising sufficient capital and finding the right sources and mechanisms of funding [289] (from investment to translational grants funding this work) will help to bridge this “valley of death” keeping innovative scientific discoveries on a forward-moving path [281, 290].

To develop funding for smooth transitions from preclinical work to clinical trials requires the following criteria to be met: (i) deriving retinal organoids using cGMP-grade hPSCs (ii) demonstrating cell replacement (rather than neuroprotective) mechanisms underlying vision improvement (iii) using “large eye” animal models of retinal degeneration (such as cats, dogs, pigs or rabbits) [291, 292] and iv) establishing FDA criteria for gene and cell therapy products for characterization of hPSC-3D retinal tissue (sterility, mycoplasma contamination, endotoxins, identity (DNA fingerprinting), karyotyping, residual contamination, viability and variability).

#### Postmarket Challenges

The path for a promising stem cell therapy to become an insurance-covered medical procedure is to find the right reimbursement path, diligently carrying out and presenting valuation analysis to public and private payers and convincingly demonstrate the value of treatment [293].

The FDA approved product Provenge (from Dendreon Inc.) was in clinical trial for prostate cancer patients, where the patients treated with Provenge had their life extended by only 4.1 months (25.8) compared to untreated patients (21.7 months). This made the reimbursement hardly feasible and ultimately caused the bankruptcy of the company [294]. However, transplanting hPSC-derived retinal tissue in patients with advanced retinal degeneration promises to improve patient’s vision and to add many Quality Adjusted Life Years [293]. Vision loss has a major impact on the average patient’s quality of life. Blindness has been ranked by World Health Organization experts in the same class of increasing disability severity (Class VI) as paraplegia [295], continuing to be on top of people’s medical concerns and fears. At the same time, covering clinical procedures based on such Regen Med therapies without bankrupting the payers requires rethinking the path of regulatory approval, reducing the operating costs, the time from preclinical R&D work to approval, and reimbursement process. Japan introduced conditional approvals for stem cell therapies in 2014, based on early phase evidence for their safety and efficacy, with performance data collection and reporting requirements and a patient co-pay structure [296]. With conditional approvals, the financial burden of the equivalent of a phase-2 or 3 trial (in such Japanese model) shifts from the private sector to health system payers, until data on safety and efficacy are collected. In phase 2 studies one can ask valuable questions, and add measures to a trial that looks at costs and potential value. Another possibility is for all involved parties including industry, government and patients pay their share equally. Clearly, new and innovative ways for reimbursement processes need to be developed to share the high cost of new therapies between the biotech, health system payers, the government and the patients. The searching for innovative cost- and risk-sharing models, based on economic sustainability, is ongoing, and adapting an RMAT mechanism for promising stem cell treatments of blindness is one step forward to test if this mechanism may work. Urgency for finding any working treatment for blindness greatly helps stem cell-based vision restoration technologies to take major advantage of this new opportunity. Whether this opportunity will stay open will depend on results of clinical trials [149, 284]. At least for using the hPSC-derived retinal tissue approach for vision treatment, the promise remains within reach and is strongly supported by 20-years of work on transplanting human fetal retinal tissue in animals and patients. The work on bioprosthetic retina will continue to evolve and merge with work on neuroprosthetic implants (which already have working reimbursement mechanisms). The present neuroprosthetic devices are functionally limited due to limitations in design, size, anticipated life span, electronics, and risks associated with the implant placement. A neuroprosthetic retina has the potential to overcome these limitations by integrating permanently into the synaptic circuitry of recipient retina. Neuroprosthetics is a growing field that has the potential to re-engineer a patient’s lost sense of sight and greatly improve his or her quality of life. Therefore, the future of commercialization of 3D retinal tissue technologies looks bright, although the search for the optimal implementation is still ongoing.

## Summary

The plasticity of hPSCs makes them an excellent tool for the pharmaceutical industry and the regenerative medicine, including vision restoration. Based on clinical trial reports and analysis of recent literature, the current most critical requirements to translating hPSC-3D retinal tissue into clinical applications are: (i) establishing robust and reproducible protocols for the efficient derivation of retinal organoids from hPSC; ii) generating retinal tissue from low-passage cGMP-grade hPSCs from stem cell banks, with normal karyotype and in cGMP-environment; (iii) producing larger pieces of retinal tissue from hPSCs for subretinal grafting along with supporting biomaterials; (iv) conducting long-term (1–2 year rather than 2–6 month-long) in vivo vision rescue experiments in large eye preclinical *rd* animal models (dog, cat, pig or rabbit), (vi) improving surgical methods of delivering retinal tissue into subretinal space, vii) using creative combinations of biomaterials to enable the survival and functional (synaptic) integration of the hPSC-retinal tissue grafts into recipient’s neural circuitry. This will enable transitioning of hPSC-3D retinal tissue technology to the clinic faster to benefit millions of people with blindness suffering from retinal degeneration.
